# Plant receptor-like kinase signaling through heterotrimeric G-proteins

**DOI:** 10.1093/jxb/eraa016

**Published:** 2020-01-13

**Authors:** Sona Pandey

**Affiliations:** 1 Donald Danforth Plant Science Center, St Louis, MO, USA; 2 University of Missouri, USA

**Keywords:** Extra-large G-proteins, G-protein activation, G-protein-coupled receptors, heterotrimeric G-proteins, receptor-like kinases, RGS proteins

## Abstract

Heterotrimeric G-proteins regulate multiple aspects of plant growth, development, and response to biotic and abiotic stresses. While the core components of heterotrimeric G-proteins and their basic biochemistry are similar in plants and metazoans, key differences exist in their regulatory mechanisms. In particular, the activation mechanisms of plant G-proteins appear diverse and may include both canonical and novel modes. Classical G-protein-coupled receptor-like proteins exist in plants and interact with Gα proteins, but their ability to activate Gα by facilitating GDP to GTP exchange has not been demonstrated. Conversely, there is genetic and functional evidence that plant G-proteins interact with the highly prevalent receptor-like kinases (RLKs) and are phosphorylated by them. This suggests the exciting scenario that in plants the G-proteins integrate RLK-dependent signal perception at the plasma membrane with downstream effectors. Because RLKs are active kinases, it is also likely that the activity of plant G-proteins is regulated via phosphorylation/dephosphorylation rather than GTP–GDP exchange as in metazoans. This review discusses our current knowledge of the possible RLK-dependent regulatory mechanisms of plant G-protein signaling in the context of several biological systems and outlines the diversity that might exist in such regulation.

## Teaser

A review of the regulation of plant G-protein signaling by receptor-like kinases, discussing their genetic, functional, and biochemical links with different G-protein components and suggesting the existence of plant-specific signaling mechanisms.

## Introduction

Heterotrimeric G-proteins are signal transducers present at the plasma membrane of eukaryotic cells. The core heterotrimeric G-protein complex (G-proteins, hereafter) comprises three dissimilar subunits, Gα, Gβ, and Gγ. Gα is the catalytically active protein of the heterotrimer, which can bind with and hydrolyse guanine (G) nucleotides. When Gα is bound to GDP, it maintains a trimeric conformation by forming a tight association with the Gβγ proteins. This is considered to be the inactive or resting stage of the signaling complex. Upon activation in response to a signal, GDP on Gα is exchanged for GTP, which causes a change in the Gα conformation, resulting in the release of Gβγ dimers. Upon dissociation, both GTP–Gα and Gβγ can interact with various effector proteins to transduce specific signals. This represents the active stage of signaling. The Gα protein also has an inherent GTPase activity, which causes the hydrolysis of bound GTP, resulting in the formation of its GDP-bound form. GDP–Gα reassociates with the Gβγ dimer, reconstituting the inactive trimer, ready for the next cycle of activation (Reed, 1990; Rodbell, 1992; Oldham and Hamm, 2008). This guanine nucleotide-dependent transition of the Gα protein between trimeric (inactive) and monomeric (active) forms allows it to act as a bimodal molecular switch, regulating multiple signaling pathways with precision and efficiency (Ross, 2008). This basic mechanism is conserved in all organisms (McCudden *et al.*, 2005; Schaap, 2005; Stateczny *et al.*, 2016; Xu *et al.*, 2016).

G-protein-dependent signaling pathways are highly prevalent in mammalian systems and are estimated to be the target of more than a third of all pharmaceutical drugs due to their role in regulating the organisms’ responses to multiple sensory signals, hormones, and neurotransmitters (Hauser et al., 2017, 2018). To respond to a variety of signals, most metazoans possess expanded G-protein networks with multiple G-protein subunits that have diverse biochemical properties and interaction specificities. For example, 23 Gα, five Gβ, and 12 Gγ proteins form the core G-protein network in humans (Cabrera-Vera *et al.*, 2003; Offermanns, 2003). In contrast, the G-protein subunit repertoire in plants is smaller, but consists of both canonical and plant-specific proteins. In plants with simpler genomes such as Arabidopsis, the G-protein core is represented by one canonical and three extra-large Gα (XLG), one Gβ, and three Gγ (two canonical and one plant-specific) proteins (Pandey, 2019). Plants with complex, polyploid genomes maintain expanded networks of G-proteins, e.g. four canonical and 12 extra-large Gα, four Gβ and 12 Gγ proteins in soybean. However, the subunit multiplicity in these plants is a result of recent genome duplications and consequently the proteins do not exhibit the structural and functional diversity seen in metazoan G-proteins. Despite their limited numbers, the roles of G-proteins in plant signaling and development are diverse. Studies in Arabidopsis, rice, maize, soybean, and a few other plant species have identified G-proteins as key modulators of growth and development. G-proteins affect fundamental cellular processes such as cell division and expansion, ion channel activities and response to all plant hormones. In addition, G-proteins regulate both biotic and abiotic stress responses of plants as well as key agronomic traits such as water and nitrogen use efficiency, seed size, and seed number per plant, thereby directly affecting yield (Botella, 2012; Stateczny *et al.*, 2016; Xu *et al.*, 2016; Wu *et al.*, 2018a; Pandey, 2019; Xu *et al.*, 2019).

Early studies of plant G-proteins were heavily influenced by the metazoan model of signaling. In fact, the plant G-proteins were identified based on sequence similarities with their metazoan homologs, and the signaling mechanisms in yeast or mammalian systems were reasonably well established before plant G-proteins were discovered. Years of studies have now confirmed that although the G-protein core subunits, the interactions between them, and their basic biochemistries are conserved across phyla, their regulatory and signaling networks are probably wired differently in plant versus metazoans (Pandey, 2019).

One of the most obvious differences is in their activation mechanism. In metazoans, G-proteins are activated by an exchange of GDP for GTP on Gα (McCudden *et al.*, 2005; Siderovski and Willard, 2005). This exchange is facilitated by G-protein-coupled receptors (GPCRs), which have seven transmembrane (7TM) domains and are localized in the plasma membrane; these are often represented by large gene families (Oldham and Hamm, 2008; Satake and Sakai, 2008; Baltoumas *et al.*, 2013; Stewart and Fisher, 2015). The human genome encodes ~800 GPCRs, which perceive diverse signals, although ligands for many remain unidentified. Plants possess proteins with similarities to mammalian GPCRs; however, their role in the activation of the G-protein cycle remains equivocal. How the plant G-protein cycle is activated continues to be one of the most enigmatic and actively pursued areas of research.

## Possible activation mechanisms of G-proteins in plants

There are three hypotheses for the activation mechanism of G-protein signaling in plants, each with some supporting evidence. The first, most conservative hypothesis is that the plant Gα proteins are activated by a classical GPCR-dependent mechanism, similar to that established for the metazoan Gα proteins. As per the metazoan paradigm, for a protein to be defined as a GPCR two criteria need to be fulfilled. It should physically interact with a Gα protein and it should act as a guanine nucleotide exchange factor (GEF), causing G-protein activation. Several proteins in plant genomes have a 7TM domain topology similar to metazoan GPCRs. Plant Gα proteins interact with many of these GPCR-like proteins (Pandey and Assmann, 2004; Gookin *et al.*, 2008; Pandey *et al.*, 2009; Tuteja, 2009; Yadav and Tuteja, 2011; Gookin and Bendtsen, 2013). Moreover, plant Gα proteins are structurally similar to their mammalian Gα homologs (Jones *et al.*, 2011) and have maintained their ability to be activated by a classical GCPR, as shown by the complementation of yeast *gpa1* mutants by soybean Gα proteins (Roy Choudhury *et al.*, 2014). Additionally, at least in the case of Arabidopsis G-protein-coupled receptor 1 (GCR1), which shows significant sequence similarity with a GPCR in *Dictyostelium*, CAR1, there is ample evidence for interaction with the Arabidopsis Gα protein (AtGPA1). AtGPA1 and GCR1 work in the same molecular-genetic pathways to regulate growth and development (Pandey and Assmann, 2004; Chakraborty et al., 2015*a*,*b*; Warpeha *et al.*, 2006).

The second criterion for GPCR identity has not been demonstrated in plants, however. None of the plant GPCR-like proteins identified to date, including GCR1, has been shown to exhibit a GEF activity, i.e. the ability to facilitate the exchange of GTP for GDP on Gα. There is a possibility that the difficulty associated with the purification and characterization of the 7TM-containing proteins, combined with the lack in plants of the sophisticated assays that exists for the mammalian G-protein signaling readout, has impeded the identification of canonical GPCRs in plants. Until such an activity is experimentally demonstrated, a classical GPCR-dependent activation of the G-protein cycle in plants remains hypothetical.

A second possibility is suggested by the unique biochemical properties of AtGPA1. Compared with non-plant systems, AtGPA1 exhibits a significantly higher rate of GTP binding *in vitro* and a rate of GTP hydrolysis slower by almost an order of magnitude than that of the slowest mammalian Gα (Johnston *et al.*, 2007; Roux *et al.*, 2011; Urano *et al.*, 2012a). If such properties are maintained *in vivo*, it may result in a situation in which AtGPA1 becomes inherently GTP-bound without the help of a GEF-activity-possessing GPCR, i.e. it is self-activated. In this case, deactivation of the active Gα would control the G-protein cycle. This has been suggested to be the mechanism during G-protein-dependent sugar signaling in Arabidopsis, which is controlled by the regulator of G-protein signaling (RGS) protein (Johnston *et al.*, 2007; Booker *et al.*, 2010; Urano *et al.*, 2012b). RGS proteins act as GTPase activity-promoting proteins (GAPs) and increase the GTPase activity of Gα proteins by at least an order of magnitude, resulting in their fast deactivation.

Although this second hypothesis explains many phenotypes of Arabidopsis *gpa1* and *rgs1* mutants, its broader applicability remains to be established. Many plants do not have an RGS protein homolog (Hackenberg *et al.*, 2017). Moreover, Gα proteins with small differences in their biochemical properties do lead to distinct plant phenotypes, necessitating a careful analysis of the extent to which the biochemical properties observed *in vitro* are relevant *in planta* (Roy Choudhury and Pandey, 2017*b*). Additionally, the biochemical properties of XLG proteins have not been characterized in detail. Based on sequence analysis, these might not have a considerable GTPase activity. The interaction of XLG proteins with RGS1 proteins has also not been unequivocally established (Urano and Jones, 2014). Since XLG proteins form the core of G-protein trimers in plants and share the Gβγ proteins with the canonical Gα proteins (Pandey *et al.*, 2008; Chakravorty *et al.*, 2015; Maruta *et al.*, 2015; Hackenberg *et al.*, 2016; Urano *et al.*, 2016), their activation/deactivation kinetics need to be considered in proposing a model based on the biochemical properties of the canonical Gα proteins. Therefore, it is unclear whether the G-protein cycle is solely regulated by RGS-mediated deactivation.

A third and potentially more likely possibility is that the plant G-proteins have entirely distinct activation mechanisms, via their interaction with the highly prevalent receptor-like kinases (RLKs). RLKs constitute a large family of receptor proteins in plants, with up to 600 members in Arabidopsis (Shiu and Bleecker, 2001; Gish and Clark, 2011). They integrate a multitude of external and endogenous cues for plant developmental and stress responses. These are plasma membrane-localized, single-pass transmembrane proteins that exhibit homology to mammalian interleukin-1 receptor-associated kinase (IRAK)/Pelle kinases (Shiu and Bleecker, 2001, 2003; Shiu *et al.*, 2004; Gish and Clark, 2011). In addition to the intracellular domain with kinase activity, plant RLKs possess an extracellular N-terminal domain, which can bind various ligands. The extracellular domain is diverse and may include leucine-rich repeats (LRR), self-incompatibility (S) domains, epidermal growth factor repeats (EGRFs), lysine motif (LysM) or lectin domains (Shiu and Bleecker, 2001, 2003; Shiu *et al.*, 2004; Gish and Clark, 2011). Some of these RLKs (e.g. BRI1) have been characterized in detail with respect to their structure, ligand binding properties, and downstream signaling pathways (Kim and Wang, 2010). In most cases, RLKs have been demonstrated to function as a protein complex comprising a receptor with ligand binding ability that usually also possesses an active kinase domain and co-receptor proteins, which may include additional RLKs and other plasma membrane-localized or cytosolic receptor-like proteins (RLPs) with or without kinase activity (Rowe and Bergmann, 2010; de Vries, 2015; Burkart and Stahl, 2017; Kelly *et al.*, 2017; Wan *et al.*, 2019). Signaling via RLKs typically involves a phosphorylation/dephosphorylation-based mechanism (Tang *et al.*, 2010; Lin *et al.*, 2013; He *et al.*, 2018).

## RLK-mediated G-protein signaling pathways

The earliest evidence for the involvement of G-proteins in RLK-mediated signaling was from genetic suppressor screens, where *AGB1* (Arabidopsis Gβ) was identified as functioning in similar (e.g. in regulation of silique length) or parallel (e.g. regulation of leaf shape) pathways with an RLK Erecta (ER) (Lease *et al.*, 2001). Further characterization of the *er* and *agb1* mutants in a necrotrophic fungal defense response confirmed the role of AGB1 in ER-dependent signaling pathways (Llorente *et al.*, 2005). A suppression screen of another RLK mutant, *bir1* (*B*AK1-*i*nteracting *r*eceptor-like kinase 1), also identified *AGB1*. Loss of function mutation in *BIR1* resulted in constitutive cell death and defense response, which was suppressed by the loss of *AGB1*, implying a genetic and functional link between these two proteins (Liu *et al.*, 2013). Incidentally, AGB1 might function downstream of multiple RLKs as it was required for resistance responses mediated by flagellin-sensitive2 (FLS2), elongation factor-TU receptor (EFR), and chitin elicitor receptor kinase1 (CERK1), three well-established RLKs in pathogen-associated molecular pattern (PAMP)-triggered immunity responses in plants (Delgado-Cerezo *et al.*, 2012; Liu *et al.*, 2013; Tunc-Ozdemir and Jones, 2017). The two Arabidopsis Gγ proteins, AGG1 and AGG2, were also involved in these genetic pathways as confirmed by the phenotypes of *agg1agg2* mutants. Several follow-up studies have confirmed the roles of the Arabidopsis Gβγ complex in multiple defense signaling pathways, corroborating the genetic interactions (Zhu *et al.*, 2009; Chakravorty *et al.*, 2012; Chen and Brandizzi, 2012; Delgado-Cerezo *et al.*, 2012; Liu *et al.*, 2013; Brenya *et al.*, 2016; Liang *et al.*, 2016; Xu *et al.*, 2019). In contrast to AGB1 and AGG1/AGG2, the canonical and extra-large Gα proteins have not been identified in genetic screens to date; however, complementary analysis such as protein–protein interaction assays or functional characterization of the *xlg* and *gpa1* mutants has suggested their roles in RLK-mediated signaling. For example, a search of the Membrane Based Interactome Database (MIND, www.associomic.org), which lists potential interactions among all Arabidopsis membrane-localized proteins, identifies several RLKs as interactors of AGB1, XLG2, XLG3, RGS1, AGG1, and AGG2 proteins. Similarly, another study using AGB1 as bait followed by co-imunoprecipitation of interacting proteins identified several RLKs as potential interaction partners of G-proteins. Of these, Feronia (Fer) has been characterized for its role in regulating stomatal phenotypes and salinity response in conjunction with G proteins (Yu and Assmann, 2018; Yu *et al.*, 2018).

## Mechanistic details of RLK-dependent regulation of the plant G-protein cycle

While genetic and functional studies have implied that plant G-proteins can interact with RLKs and are functionally linked, the mechanistic details of such interactions have remained largely unknown until recently. A logical expectation is that the interaction between a plasma membrane-localized receptor and the members of the G-protein complex would activate the G-protein cycle to transduce the signal. Because RLKs are active kinases and their self-activation as well as signal transduction ability depends on their kinase activity, a simplistic hypothesis is that RLKs would control G-protein signaling by phosphorylating specific G-protein components. Phosphorylation-dependent regulation of G-protein signaling has already been reported in mammalian and yeast systems. A recent review discusses in exquisite details the phosphorylation of specific G-protein subunits, the potential effects of such phosphorylations on their structure/function and known roles of such phosphorylation in controlling specific signal transduction pathways in yeast, humans, and plants (Chakravorty and Assmann, 2018). Furthermore, most plant G-protein subunits, including Gα, XLGs, Gβ, Gγ, and RGS proteins, have been identified as phosphoproteins in large-scale, non-targeted proteomics experiments, although a direct association between the kinases that phosphorylate them (which may also be proteins other than RLKs) is sparse (Chakravorty and Assmann, 2018). The majority of information is available for the RGS protein phosphorylation, which has emerged as a key regulatory mechanism in plant G-protein signaling. Plant RGS proteins are unique due to the presence of a seven transmembrane receptor-like domain, which allows for their plasma membrane tethering, and a C-terminal catalytically active RGS domain. Almost all phosphoamino acids identified to date map to the C-terminal region of RGS proteins.

Intriguingly, RGS is phosphorylated by a variety of kinases, including RLKs, but there seem to be certain ‘hotspots’ where most phosphorylations have been observed. For example, the Arabidopsis RGS1 is phosphorylated by With No Lysine 8 (WNK8), by Open stomata 1 (OST1), BRI1 receptor like 3 (BRL3), BRI1 associated receptor kinase 1 (BAK1), and Botrytis induced kinase 1 (BIK1) during sugar signaling or immune signaling (Urano *et al.*, 2012b; Liang *et al.*, 2016; Tunc-Ozdemir *et al.*, 2016; Tunc-Ozdemir and Jones, 2017; Liang *et al.*, 2018). In most of these cases, the phosphorylation occurs at the Ser-428/Ser-435/Ser-436 site, which may result in its endocytosis in response to specific signals. Alternatively, the phosphorylations of these same amino acids by PAMP receptors such as FLS2, ERF, or LYK5 affect the interaction of RGS1 with specific G-proteins, thus affecting signaling Urano *et al.*, 2012b; Liang *et al.*, 2016; Tunc-Ozdemir *et al.*, 2016; Tunc-Ozdemir and Jones, 2017; Liang *et al.*, 2018). During soybean nodulation, GmRGS proteins are phosphorylated at several residues including Ser-428 and Ser-437, which results in RGS activation and potentially more efficient deactivation of Gα proteins. However, these phosphorylations did not alter RGS localization or its ability to interact with the Gα proteins (Roy Choudhury and Pandey, 2015). These observations offer a glimpse of diverse effects of phosphorylation on G-protein signaling. Although the *in vivo* data are still lacking in some of these cases or the kinases that can directly phosphorylate G-proteins remain to be characterized, the specific details that have emerged from a few signaling systems in which G-protein–RLK interactions have been demonstrated suggest both expected and novel mechanisms.

### Plant–microbe interaction

One of the most extensively characterized roles of G-proteins in RLK-dependent signaling is during plant–microbe interactions. As discussed previously, genetic studies have long identified the roles of G-proteins in conjunction with multiple defense signaling-related RLKs. Two examples for which mechanistic details have become available are the defense response in Arabidopsis and nodule formation in soybean.

#### Defense response in Arabidopsis

**Fig. 1.**
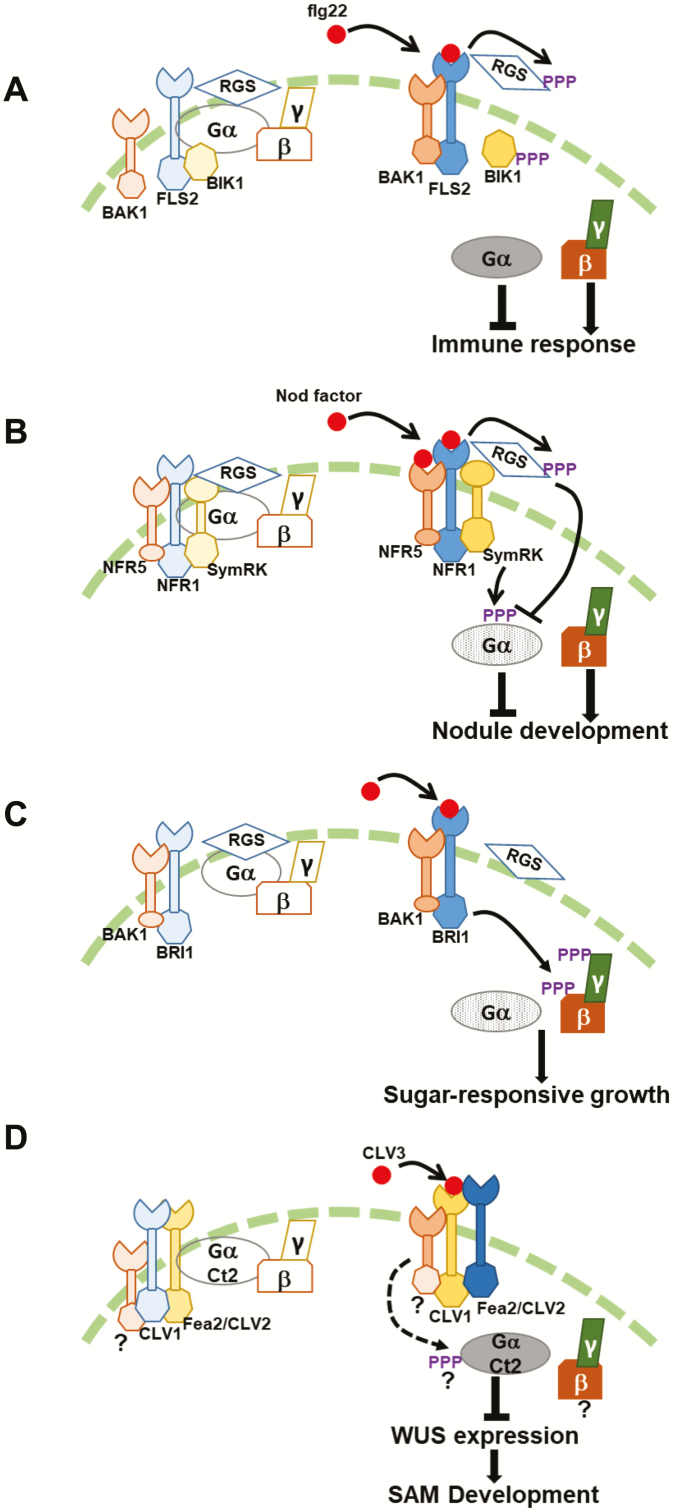
Possible mechanisms of RLK-mediated control of G-protein activation. (A) During immune response, receptor-mediated phosphorylation of RGS protein causes its dissociation from the G-protein complex, allowing for the heterotrimer dissociation. The G-proteins are active due to the spontaneous GTP-binding ability of the Gα proteins. Gα represents both canonical and XLG proteins. (B) During nodule development, NFR1 proteins phosphorylate RGS proteins and SymRKs phosphorylate Gα proteins. RGS phosphorylation deactivates Gα. In addition, phosphorylated Gα fails to interact with the Gβγ dimer. This dual regulation causes inactivation of the negative regulator and availability of the positive regulator to transduce the signal. (C) During sugar-responsive growth and development, the BRI1–BAK1 receptor kinase pairs phosphorylate the Gβ and Gγ proteins, potentially resulting in their dissociation from the heterotrimer, and thus activation of the G-protein cycle. (D) During SAM development in maize (and in Arabidopsis), RLKs and RLPs act via G-proteins, but the effect of RLKs on G-proteins is currently not known. Light and dark colors indicate inactive and active states, respectively, of specific signaling components.

The role of heterotrimeric G-proteins in plant immune signaling has been reviewed recently (Zhong *et al.*, 2019), so I focus here only on RLK-dependent activation of G-protein signaling. AtGPA1 has an unusually high rate of GTP binding coupled with an exceptionally slow rate of GTP hydrolysis, suggesting that it is self-activated, i.e. it does not require a GDP to GTP exchange for activation. In this situation, the trimeric, inactive G-protein complex exists primarily due to the GAP activity of the RGS proteins. RGS proteins, by promoting GTP hydrolysis, help generate the GDP-bound form of Gα, which remains associated with Gβγ. Protein–protein interactions and biochemical activity assays have demonstrated that in Arabidopsis during the resting phase of immune signaling, the G-protein trimeric complex comprised of GPA1 (or XLG2 or XLG3), AGB1, and AGG1 (or AGG2) is associated with the FLS2–BAK1–BIK1 receptor complex at the plasma membrane (Liang *et al.*, 2016; He *et al.*, 2018; Xu *et al.*, 2019). RGS1 is also a part of the complex as it interacts with both GPA1 and the receptors. Ligand (flg22) binding activates the FLS2 receptor, which causes the activation of a downstream kinase, BIK1. BIK1 phosphorylates RGS1 at multiple sites. In a parallel mechanism, the co-receptor BAK1 also phosphorylates RGS1. Phosphorylated RGS1 dissociates from the G-protein–receptor complex and possibly internalizes or is subjected to degradation. Release of RGS1 sets free the G-protein complex, which, due to the self-activation of Gα, dissociates from the Gβγ dimer. Both these entities can transduce the signal when freed (Liang *et al.*, 2018; Liang and Zhou, 2018; Wang *et al.*, 2018) (Fig. 1A). These reports also demonstrated that different types of Gα proteins might control specific aspects of plant immunity and defense responses. For example, stomatal immunity was proposed to depend on the GPA1–Gβγ heterotrimer whereas the immune responses mediated via mesophyll cells primarily used the XLG–Gβγ heterotrimer (He *et al.*, 2018; Liang *et al.*, 2018; Wang *et al.*, 2018).

This proposed mechanism shows both similarities to and difference from the metazoan paradigm of G-protein activation. On the one hand, it shows the receptor-dependent dissociation of the trimeric, inactive complex to release the active proteins, similar to the classical G-protein activation mechanism. On the other hand, it also demonstrates that, in contrast to the metazoan systems, activation is not via the GEF activity of a receptor but via the abolition of the GAP activity of RGS, which in the absence of a ligand represses the constitutively active Gα (Fig. 1A).

This mechanism is attractive, because BAK1 and BIK1 receptors are a part of multiple RLK complexes involved in regulation of growth and development as well as immune signaling responses (Roux *et al.*, 2011; Sun *et al.*, 2013; Prince *et al.*, 2014; Yeh *et al.*, 2016; Imkampe *et al.*, 2017; Peng *et al.*, 2018; Xue *et al.*, 2019). Therefore, a similar mechanism might operate in additional signaling pathways, implying its broader applicability. However, several issues remain to be addressed especially in the context of XLG and RGS proteins. The GTP-binding and GTPase activity of XLG proteins are not well characterized and it is not known if these are also self-activated as suggested for the canonical Gα proteins. Moreover, whether XLG proteins interact with RGS proteins or whether they possess GTPase activity that can be promoted by RGS proteins remains an open question. Additionally, G-proteins are known to affect defense responses in plants such as rice, which does not possess an RGS encoding gene in its genome (Hackenberg *et al.*, 2017). How the RGS-dependent mechanism of RLK/G-protein signaling would operate in plants with no known RGS homologs will be an interesting area of future research.

#### Nodulation in soybeans

G-proteins and RLK-mediated signaling has been found to be involved in symbiotic nitrogen fixation in soybean. N_2_ fixation is an exquisitely controlled process regulated by multiple interconnected signaling networks, which coordinate plant–microbe interaction resulting in altered growth, development, and nodule organogenesis (Desbrosses and Stougaard, 2011; Oldroyd *et al.*, 2011; Antolín-Llovera *et al.*, 2012). Nodulation starts with the secretion of rhizobial nodulation factors (Nod factors, NF), which are perceived by the plasma membrane-localized RLKs containing the LysM motifs (Radutoiu *et al.*, 2003; Antolín-Llovera *et al.*, 2012; Broghammer *et al.*, 2012). Pharmacological studies suggested roles for G-proteins in signaling during nodulation (Kelly and Irving, 2003; Sun *et al.*, 2007; De Los Santos-Briones *et al.*, 2009), which was confirmed later using soybean hairy roots expressing altered levels of specific G-protein components. In soybean, Gα proteins are negative regulators whereas the Gβ and Gγ proteins are positive regulators of nodule formation, i.e. suppression of Gα or Gβ by RNAi resulted in higher or lower nodule number per root, respectively. Suppression of RGS proteins, which are deactivators of the G-protein cycle, resulted in phenotypes opposite to those with the suppression of the Gα protein, i.e. fewer nodules per root (Roy Choudhury and Pandey, 2013).

In soybean, NFs are perceived by Nod factor receptor 1 (NFR1) and NFR5 protein pairs. NFR1 but not NFR5 possesses an active kinase domain although both bind NFs (Madsen *et al.*, 2003; Indrasumunar *et al.*, 2011). Several downstream components of signaling during nodulation, especially those related to nuclear calcium oscillation and transcription factors, are well established, but the details of the proteins acting directly downstream of NF receptors are less obvious (Desbrosses and Stougaard, 2011; Oldroyd *et al.*, 2011). The soybean Gα and RGS proteins interact with NFR1 proteins. NFR1 phosphorylates RGS proteins. Phosphorylation of RGS proteins increases their GAP activity, which deactivates the G-protein cycle by generating inactive Gα. As Gα proteins are negative regulators of nodule formation, their receptor-dependent deactivation results in successful nodulation (Roy Choudhury and Pandey, 2015). This model was further corroborated by overexpressing a phosphomimic version of the RGS protein in the *nod49* mutants. These mutants do not have a functional NFR1α protein, and do not develop nodules. However, the expression of a phosphomimic version of RGS protein in *nod49* mutant background restores nodule formation, at least partially (Roy Choudhury and Pandey, 2015). This supports the notion that at least one role of NFR1 is to phosphorylate RGS proteins, which allow successful nodulation by deactivating the Gα proteins (Fig. 1B).

While this model explained how the Gα proteins are maintained in their inactive conformation during nodulation, the RLK-dependent regulation was indirect. However, our recent data point to an additional layer of control by another RLK, which directly affects Gα. Symbiosis-related receptor kinase (SymRK or NORK) is another RLK that forms an integral part of the nodule receptor complex in soybean. SymRKs interact with the Gα proteins and importantly phosphorylate them at multiple sites (Roy Choudhury and Pandey, 2019, Preprint). Two of the amino acids, which are phosphorylated by SymRK, are located in the highly conserved GTP-binding pocket of the Gα proteins. As expected, phosphorylation of these amino acids interferes with the ability of Gα to bind GTP, i.e. once phosphorylated, the Gα proteins become biochemically inactive, and cannot bind and (consequently) hydrolyse GTP (Fig. 1B).

These results were counter-intuitive because the expectation, based on the mammalian signaling paradigm, is that receptor-mediated phosphorylation would likely activate the Gα protein. However, further examination of the nodulation signaling pathway, based on the expression of native, phospho-dead and phospho-mimetic versions of Gα proteins in soybean hairy roots, suggested a unique mode of regulation independent of the biochemical activity of the Gα protein. While at the biochemical level the phospho-mimetic and phospho-dead versions of Gα exhibited identical properties, i.e. neither version exhibits GTP binding or hydrolysis due to alteration of the critical amino acids in the active site, their effects on nodule formation were distinct. Follow-up experiments suggested that the effect of SymRK-mediated phosphorylation was facilitated via changes in the interaction specificity of Gα protein. Yeast-based and *in planta* protein–protein interaction assays show that the phospho-mimetic Gα proteins (but not the phospho-dead versions) fail to interact with Gβγ dimers, although their ability to interact with the RGS proteins remains unchanged. Based on these data, the following model of RLK-dependent regulation of G-protein signaling during nodulation emerges (Fig. 1B). A receptor protein complex (i.e. NFR1–NFR5–SymRK) interacts with and phosphorylates different G-protein components to exert a two-pronged effect on the G-protein cycle. Phosphorylation of RGS by NFR1 maintains Gα in the inactive form whereas phosphorylation of Gα by SymRK abolishes its interaction with Gβγ. Such a scenario would allow for the inactivation of the negative regulator (Gα) and signaling by the positive regulators (Gβγ), resulting in successful nodulation.

While this mechanism does not exactly address the ‘activation’ of G proteins, it certainly uncovers a yet-unexplored signaling scheme via plant G-proteins and RLKs in which the trimer is dissociated as a result of receptor activation. The roles of XLGs have not been examined in nodule signaling and development to date. However, the amino acids, which are phosphorylated in the active site of canonical Gα, are conserved in the GTP-binding pocket of XLG proteins. XLG proteins interact with RLKs and are known phosphoproteins. Therefore, it may represent an additional regulatory mechanism for plant G-protein signaling and warrants further exploration.

### Plant development

The roles of G-proteins in plant development have been studied in mechanistic detail. G-proteins are known to control development during multiple stages of plant growth. In Arabidopsis, the G-protein mutants exhibit rounder and crinkled leaves, altered rosette size and root mass, and differences in the size and shape of various reproductive organs when compared with the wild-type plants (Lease *et al.*, 2001; Ullah et al., 2001, 2003; Pandey and Vijayakumar, 2018; Pandey, 2019). In all monocot species studied to date, the developmental phenotypes of G-protein mutants are even more striking; Gα mutants are dwarf, with severely altered aboveground architecture, whereas a complete loss of the Gβ or XLG proteins in rice and maize results in seedling lethality (Iwasaki *et al.*, 1997; Fujisawa *et al.*, 1999; Perfus-Barbeoch *et al.*, 2004; Utsunomiya *et al.*, 2011; Bommert *et al.*, 2013; Urano *et al.*, 2015; Wu *et al.*, 2018a). In both maize and Arabidopsis, there is evidence for the role of RLKs in G-protein-dependent regulation of plant development.

#### Shoot apical meristem development in maize

Shoot apical meristem (SAM) development is an exquisitely controlled pathway that maintains a continuous supply of stem cells throughout the life of the plant. A homeobox transcription factor, Wuschel (WUS), and a Clavata (CLV) receptor-kinase module form the central regulatory pathway during SAM development (Somssich *et al.*, 2016). WUS promotes stem cell proliferation whereas the CLV pathway controls cell differentiation, working in a negative feedback loop. In plants, the CLV pathway typically consists of CLV1, an RLK, CLV2, an RLP, and CLV3, which is a small peptide that is the ligand for CLV1. The role of G-proteins has been shown in SAM development by demonstrating a genetic and functional interaction between maize CLV2 homolog (Fea2) and the canonical Gα (Ct2) (Bommert *et al.*, 2013). Reduced expression of either *Ct2* or *Fea2* resulted in increased SAM size, which was similar to what was observed in a double mutant, suggesting the two genes function in the same pathway. Ct2 transmits the CLV-dependent signal as the *ct2* mutants are significantly less sensitive to the inhibitory effects of CLV3 peptides on SAM development (Bommert *et al.*, 2013) (Fig. 1C). CLV/Ct2 signaling does involve the classical G-protein cycle because it is affected by the GTP binding and hydrolysis activity of Ct2. Introduction of a constitutively active version of Ct2, which exhibits no GTPase activity (Ct2^CA^), in the *ct2* mutant background results in partial complement of the mutant phenotype; a *ct2:Ct2*^*CA*^ plant shows the phenotypes of a weak allele of *ct2*. The extra-large Gα proteins of maize also regulate SAM size, both with Ct2 and independent of it. Maize has three genes encoding XLGs. The *xlg* triple mutants of maize survive only until the young seedling stage, but do not exhibit any difference in SAM development. However, when any two of the *XLG* genes are knocked-down in a *ct2* mutant background, the SAM is significantly larger (Je *et al.*, 2018; Wu et al., 2018*a*,*b*).

While these genetic data confirm regulation of SAM development in maize via an RLK-dependent, G-protein-coupled pathway, the downstream signaling events are not yet defined. The effects of RLK interaction on Gα proteins are unknown, nor is it known how the interaction affects or integrates with other signaling modules in SAM development.

### Shoot apical meristem development in Arabidopsis

The CLV/WUS pathway controls SAM development in Arabidopsis as well, and there is some evidence for the involvement of G-proteins, but somewhat different from what has been described for maize. In Arabidopsis, the Gβ mutant (*agb1*) and the mutants lacking all four Gα genes (*gpa1.xlg1.xlg2.xlg3* quadruple mutant) exhibit an enlarged SAM but not the single *gpa1* mutants or the *xlg1.xlg2.xlg3* triple mutants, suggesting that different Gα proteins have redundant roles in affecting SAM development with the sole Gβ gene (Urano *et al.*, 2016). A genetic screen for enhancers of the *clv2* mutant identified *AGB1* (Ishida *et al.*, 2014). Similar to what is reported for maize Gα (*ct2*), the *agb1* mutants also exhibited reduced sensitivity to CLV3 peptide, implying that the G-proteins are signaling via the classical CLV module. A receptor-like kinase, RPK2, which is a component of the CLV signaling module in Arabidopsis, interacts with AGB1. However, the epistatic interactions between *RPK2*, *CLV2*, *CLV1*, and *AGB1* are not clear. The *agb1.clv2*, *agb1.clv1* or *agb1.rpk2* mutants show either additive or similar phenotypes when examining different traits regulated by the CLV pathway, implying partial independent regulation (Ishida *et al.*, 2014). Additionally, the details of the effects of CLV2 or RPK2 on G-protein activity or interaction have not been explored yet.

#### Sugar-responsive growth in Arabidopsis

An interaction between the well-known brassinosteroid receptor and co-receptor BRI1–BAK1 complex with the G-protein subunits to control sugar-responsive growth and development in Arabidopsis is another example of RLK-mediated regulation of G-protein signaling (Peng *et al.*, 2018). Although the mechanistic details of how these receptors affect G-protein signaling or cycle remain largely unknown, it has been demonstrated that the G-proteins work in the same genetic pathway as BRI1–BAK1 receptors, and the receptors interact with Gβγ proteins. Moreover, the BRI1 kinase domain phosphorylates AGB1 and AGG3 *in vitro*, and the phosphorylation does affect sugar-responsive growth (Peng *et al.*, 2018). No direct interaction between GPA1 and BRI1–BAK1 was observed but the authors speculate that BRI1–BAK1-dependent phosphorylation of the AGB1–AGG3 dimer may cause their dissociation from GPA1, thus activating signaling (Fig. 1D). Further validation of such a model will be important to establish it as a unique G-protein activation mechanism.

Additional examples of involvement of RLKs during G-protein signaling include the interaction of Feronia (Fer) with the Arabidopsis Gβ protein during the control of stomatal aperture and during salinity response (Yu and Assmann, 2018; Yu *et al.*, 2018), and ZAR1 (zygotic arrest 1) and AGB1 interaction during asymmetric cell division in zygotes (Yu *et al.*, 2016). However, the mechanistic details of these physiological observations or genetic interactions remain to be established.

## Perspectives and future directions

Overall, these examples demonstrate that plant G-protein signaling can be regulated by RLKs, thus presenting an exciting opportunity to elucidate the individual roles of RLKs and G-proteins and the connections between them. This discussion also provides a glimpse of the varied modes of regulation of the G-protein cycle in plants, while not excluding the possibility that the established mammalian paradigm may also exist. Many other receptor-like proteins, such as GCR1, MLOs, and GTG proteins, participate in G-protein dependent pathways and may have overlapping roles with RLKs (Pandey and Assmann, 2004; Pandey *et al.*, 2009; Lorek *et al.*, 2013). Similarly, the plant G-protein cycle is also regulated by various phospholipases and lipid signaling components, some of which seem to work upstream of G-proteins (Hong *et al.*, 2016; Roy Choudhury and Pandey, 2016, 2017*a*). It may be that the G-protein signaling in plants does not operate as a simple combination of activation (on) and deactivation (off) stages, but as a complex and interconnected set of such steps, each of which enables or disables particular interactions. It can be envisioned that by having multiple, interconnected modules each with its own on–off states, a specific combinatorial sets of ‘on’ and ‘off’ states results in different signaling outputs. Given the smaller repertoire of G-proteins in plants, but their involvement in almost all aspects of plant growth and development, such diverse mechanisms may be critical for effective signaling. Additionally, such multi-faceted regulation of G-proteins may also provide extreme flexibility to the G-protein networks. Signaling networks have evolved to be highly flexible to suit the sedentary life style of plants. Further research will certainly enrich the plant G-protein signaling field, but may also provide unique perspectives on the non-plant G-protein signaling fields.
